# AI revolution in healthcare and medicine and the (re-)emergence of inequalities and disadvantages for ageing population

**DOI:** 10.3389/fsoc.2022.1038854

**Published:** 2023-01-23

**Authors:** Justyna Stypińska, Annette Franke

**Affiliations:** ^1^Department of Sociology, Institute of East European Studies, Free University of Berlin, Berlin, Germany; ^2^European New School of Digital Studies, Viadrina University, Frankfurt (Oder), Brandenburg, Germany; ^3^Department of Social Work, Evangelische Hochschule Ludwigsburg, Ludwigsburg, Baden-Württemberg, Germany

**Keywords:** artificial intelligence, automatic decision making, health care, ageing population, ageism

## Abstract

AI systems in medicine and healthcare are being extensively explored in prevention, diagnosis, novel drug designs and after-care. The application of AI technology in healthcare systems promises impressive outcomes such as equalising healthcare, reducing mortality rate and human error, reducing medical costs, as well as reducing reliance on social services. In the light of the WHO “Decade of Healthy Ageing”, AI applications are designed as digital innovations to support the quality of life for older persons. However, the emergence of evidence of different types of algorithmic bias in AI applications, ageism in the use of digital devices and platforms, as well as age bias in digital data suggests that the use of AI might have discriminatory effects on older population or even cause harm. This paper addresses the issue of age biases and age discrimination in AI applications in medicine and healthcare systems and try to identify main challenges in this area. It will reflect on the potential of AI applications to amplify the already existing health inequalities by discussing two levels where potential negative impact of AI on age inequalities might be observed. Firstly, we will address the technical level of age bias in algorithms and digital datasets (especially health data). Secondly, we will discuss the potential disparate outcomes of automatic decision-making systems (ADMs) used in healthcare on the older population. These examples will demonstrate, although only partially, how AI systems may create new structures of age inequalities and novel dimensions of exclusion in healthcare and medicine.

## 1. Introduction

Demographic ageing is often associated with cost-intensive multimorbidity, a shortage of skilled workers that is already noticeable in care and medicine and changing expectations and demands of older people for adequate health care. Digitalisation of healthcare, and particularly the recent developments in artificial intelligence (AI) for health seem to promise a way out of this dilemma and an important relief, postponing or even avoiding cost-intensive inpatient care help. The application of AI technology in healthcare systems promises impressive outcomes such as equalising healthcare, reducing mortality rate and human error, reducing medical costs, as well as decreasing reliance on social services (Davenport and Kalakota, 2019; Mukaetova-Ladinska et al., 2020). According to experts, AI is expected to make a profound impact on healthcare and ageing research (Zhavoronkov et al., 2019). The AI in healthcare was estimated to generate 6.9 billion USD in 2021 and is expected to reach 67.4 billion USD by 2027 (Markets and Markets, 2021) and is certainly one of the most lucrative and investment-intense areas of AI deployment.

However, the emergence of evidence of algorithmic bias in variety of AI applications (Buolamwini and Gebru, 2018; Díaz et al., 2018), ageism in the use of digital devices and platforms (Rosales and Fernández-Ardèvol, 2020), as well as age bias in digital data suggests that the AI systems might also have discriminatory effects on older population or even cause harm (Chu et al., 2022; Stypinska, 2022). Additionally, AI algorithms are being developed from the current state of health care data and are thus embedded in the context of deprivation and health inequality (Nordling, 2019). This was particularly visible during the COVID-19 pandemic where the socioeconomic factors proved to be responsible for higher rates of morbidity and severity of disease (Ahmed et al., 2020). Moreover, Peine et al. observed that “the global pandemic has worked as a pressure cooker that has produced new configurations of old ageist and gendered stereotypes of age and ageing as problems, in which technology is seen as a solution” (Peine et al., 2021, p. 3).

In the light of the WHO “Decade of Healthy Ageing (2021–2030)”, AI applications are also designed as digital innovations to support the quality of life for older persons. The digitalisation of health care work and the inclusion of various forms of AI in health care change the justifications, legitimation and knowledge base for the transformations of care associated with mechanisation. Questions about what can be considered appropriate and what rules the use of technology, equipment and medication should follow in health care arrangements are currently being renegotiated. This is shown, for example, by the discussion that the German Ethics Council began in 2019 on the use of care robots and the associated relativisation of personal contact and the hybridisation of care relationships (German Ethics Council, 2020).

Moreover, the European Commission's proposal for an Artificial Intelligence (AI) Act has been the topic of a heated debates since its launch in April 2021. Critical researchers are worried that the that the AI Act neglects the risks health AI pose to patients' health and fundamental rights, in particular the rights to access to healthcare, non-discrimination and privacy (Kolfschooten and Oirschot, 2022). Yet, the AI Act does not specifically identify health AI as “high risk” and does not provide solutions for its key risks, as is the case in other areas of AI application (Commission, 2021). Nevertheless, the critics point to four reasons why AI for health deserves special consideration: (1) people's health is at stake, (2) people are in a vulnerable position when in need of healthcare, (3) the collection of health data has dramatically increased in recent times and (4) health data is historically littered with bias. Because of these characteristics, health AI faces unique risks that need to be specifically addressed in the AI Act (Kolfschooten and Oirschot, 2022).

When justifying and developing criteria and standards for AI, it is also important to consider that the use of technology is culturally variable as well as dependent on the environment, age and gender. People vary with age as well as with the requirements and the extent of the care work—in the case of severely disabled people as opposed to in geriatric long-term care. This indicates that AI systems form a large and heterogeneous field of social transformation, in which various participants and those affected carry out many different activities with diverse objectives. In addition, the research and application field are broadly theoretically framed and reflected.

This necessitates some systematic considerations on the range of the subject area and to look at them to see which ethical questions can be identified more precisely in the respective AI supported health care services. This question or perspective is of fundamental importance since social inequality and ageism is present in every form and variant in the health care system and its further automatisation through AI systems might lead to intensification thereof. This paper discusses the potential risk of re(emerging) inequalities and rise in age discrimination as a result of deployment of AI systems in health care sector. Ageism in the field of AI can manifest it multiple forms, from technical bias of algorithms and datasets, to ageism in discourses and narratives about older persons, to exclusion of older adults as users (Stypinska, 2022). In this perspective paper we look at the risks from two angles—(1) the technical: risk related to biases in digital datasets and algorithms, and (2) risk of discrimination from automated decision-making systems (ADMs) being increasingly used in the health care and health insurance systems.

## 2. Age bias in AI

The concern about bias in AI systems is today higher than ever. The common expression “garbage in, garbage out” (Mittelstadt et al., 2016) used to refer to the poor outcomes of AI models when the data they learn from are of poor quality alerts us to the significance of the data gathering and mining practises. “Bias in, bias out” is another catchphrase used to high-light concerns about the fact that data driven AI models make inferences by finding “patterns” from the data they analyse (Wu et al., 2022). Recent analysis shows also that age-biased samples and biased tools used for constructing algorithms tend to exclude the habits, interests and values of older people what contributes to strengthening already existing structural ageism (Rosales and Fernández-Ardèvol, 2019). Studies of age bias in machine learning are still rare, but persistently show that age bias exists in sentiment analysis models (Díaz et al., 2018), face recognition systems using advanced deep learning techniques (Meade et al., 2021), in emotion recognition systems (Kim et al., 2021), as well as in speech recognition systems (Werner et al., 2019).

The results of analysis of face recognition systems show that age estimation was generally performing poor on older age groups (60+), an effect which was compounded by gender and race (Meade et al., 2021). Analysis of software detecting emotions showed that older adults had the lowest classification accuracy scores for each of the four assessed algorithms while young adults had the highest across the board (Kim et al., 2021). Additionally, some algorithms suggest improved performance over time for certain subgroups, specifically gender subgroups. However, for age groups no such impact was observed—the differences in accuracy between the younger and older groups continued to persist throughout the years. The authors expressed conviction that training datasets are skewed towards younger adults, causing a representation bias, amongst other societal root causes (Kim et al., 2021). Moreover, automated speech recognition (ASR) systems are an example of AI technology that is increasingly present in daily life, for instance in the development of virtual assistants. However, age-related physical changes may alter speech production and limit the effectiveness of ASR systems for older individuals. Evaluation of several automated speech recognition systems confirmed previous research that has suggested that those systems have more difficulty in recognising the speech of older adults (Werner et al., 2019).

Furthermore, many of those systems use biometric data, which are “personal data resulting from specific technical processing relating to the physical, physiological, or behavioural characteristics of a natural person” (GDPR, art. 4). Most common applications are facial recognition, fingerprint recognition, voice/speech recognition. The biometric technology has potential of impacting the older persons in more direct way due to the way biological ageing impacts and changes bodily functions in older age. Touch, image, speech, and body language will all be impacted due to ageing processes but can also be impaired in groups of people with disabilities (Zhou and Gao, 2021). For instance, risk can relate to the age-linked fading away of fingerprints impacting the accuracy of their recognition (Rosales and Fernández-Ardèvol, 2020). Hence, the use of biometric data in healthcare applications and systems can pose an additional risk for older adults.

## 3. The sources of bias and the problem with (health) data

The sources of bias in AI systems are manifold. There are three points in the machine learning pipeline where bias can originate: during the data collection and pre-processing; during the selection and creation of models; and when implementing results (de Alford et al., 2020). For instance, data annotation—a practise necessary to deploy supervised machine learning, has been found to create stereotypical images of older persons suggesting ageism of the annotators (Crawford and Paglen, 2019). Furthermore, the machine learning algorithms are no more than advanced classification systems based on variety of classification measures, which inherently contain moral standards, where each standard and category valorises some point of view and silences another. To classify is human, but each classification and standard give advantage or they give suffering to a certain group or individual (Bowker and Star, 2000). Bias in AI is a reproduction of social biases and stereotypes present in data, as well as individual prejudices and stereotypes of developers of AI technology. The following section zooms on the way bias operates in the digital data sets and the challenges of health data for ageing population.

The functioning of modern-day AI systems is inherently dependent on the data they deploy. Data being the “new oil” of the modern economy (Sadowski, 2020) lead to the phenomenon of datafication of our everyday lives, homes, health, and (ageing) bodies (Lupton, 2016; Ruckenstein and Schüll, 2017; Katz and Marshall, 2018). The use of data for development of AI for health is particularly complex since the increased use and sharing of health data threatens privacy and data protection rights (Kolfschooten and Oirschot, 2022). Health data are the most intimate and sensitive data which can be obtained many ways from standardised clinical trials or from public healthcare infrastructure, but can also be inferred indirectly from e.g., web browsing or use of medical and healthcare apps and devices (Gangadharan et al., 2014). The privacy protection of health data provides a big concern for individuals and the medical professionals, but also for developers of AI systems for health. Moreover, the complexity of the advanced AI systems and their data architecture produces what Malgieri and Niklas called “vulnerable data subject” (Malgieri and Niklas, 2020). The authors explain that “involving vulnerability as a ‘heuristic tool' could emphasise existing inequalities between different data subjects and specify in a more systematic and consolidated way that the exercise of data rights is conditioned by many factors such as health, age, gender or social status” (Malgieri and Niklas, 2020). Hence, certain socio—demographic groups, such as children, older persons, persons with chronic diseases or disability, lower socio-economic status are particularly at risk of not being able to exercise their right to data privacy and protection.

The proper representation of population of older adults in different datasets and data approaches has already been identified as one of the challenges for development of fair and age-inclusive AI systems (Rosales and Fernández-Ardèvol, 2019; Sourbati and Behrendt, 2020). Data, as well as the data we lack shape the opportunities for inclusion in later life (Sourbati and Behrendt, 2020). In terms of health data, analysing the situation of older patients during the pandemic time UN identified “flagrant lack of data on older persons”, caused by inappropriate data collection methodologies or by plainly excluding those over 50 or 60 years of age from health surveys (UN, 2020). In the clinical setting, research on the application of AI systems for health concerns of older adults is performed on small samples and does not offer conditions for replication (Mukaetova-Ladinska et al., 2020). The adequate representation of older population in datasets used for training AI models might be further disturbed by data sources the use of data such as smartphones, medical, health and wellness apps (Katz and Marshall, 2018) and other IoT (Internet of Things) devices, which generate detailed logs of health related activities. One of the challenges of these datasets is their limitation to the already relatively healthy, well-off and prosperous older adults who have access and knowledge, or sometimes adequate support, to use these devices (Rosales and Fernández-Ardèvol, 2020). The class, gender and economic status play a decisive role in the distribution in access to digital technology and thus the data generated is skewed, further marginalising those already at the risk of exclusion (Chu et al., 2022). Hence, an essential question arises: what happens when datasets deployed for medical AI are non-representative, incomplete or of low-quality? In case of AI models for health, biases in the training data can lead to discrimination and individual injury or even death (Kolfschooten and Oirschot, 2022).

## 4. Automatic decision-making systems (ADMs) in healthcare

Automated decision making systems (ADMs) is a term which addresses the use of algorithms for decision-making support of human decision- makers and the automated execution of decisions, although these are not always clearly differentiated from each other (Orwat, 2020). These systems can have purpose of predicting, identifying, detecting, and targeting individuals or communities. ADMs are being increasingly used by private companies (e.g., in recruitment and personnel management) and public sectors (health care, education, social services, law enforcement) (Mittelstadt et al., 2016; Reisman et al., 2018; Orwat, 2020). In healthcare and medicine ADMs are predominantly used as an instrument in diagnostics, for therapy decisions, and for the allocation of resources in the health sector (Algorithm Watch, 2019). Advocates for the use of ADMs view them as a value-neutral, objective and apolitical cure for bias and discrimination where everyone is treated equally, however, a similarly large body of evidence suggests that those systems can have discriminatory effects on those already marginalised, such as low income groups, persons with disabilities, persons with mental illnesses, the unemployed, or the homeless (Monteith and Glenn, 2016; Eubanks, 2018; Reisman et al., 2018; Chiusi et al., 2020; Heinrichs, 2022).

Documentation of severe social and personal consequences for individuals wronged by the outputs of such systems have raised questions about their fairness and even legality (Richardson, 2019). The existing research has shown that AI-driven ADMs are subject to, or may themselves cause, bias and discrimination that may exacerbate existing health inequity among racial and ethnicity groups (Leslie et al., 2021). Through probabilistic predictions based on assumptions these systems perform a type of “social sorting” (Hogle, 2016) which might introduce new categories of people and illness and reinforce old beliefs about social differences, which ultimately might lead to worsening of already existing health disparities and access to treatments. ADM systems deployed for facilitating a more efficient distribution of resources in the health sector (e.g., systems used for allocation of organs for transplantation) or by health insurance companies to calculate the individual risk and adapt insurance costs were also reported to have severe consequences. The devastating effects of the use of ADMs on the health outcomes of members of marginalised groups was documented by Virginia Eubanks, a researcher from Albany University in the USA, who depicted how the automatised withdrawals and denials of healthcare services lead to tragic consequences, including loss of life (Eubanks, 2018). Similar observations were made by Cathy O'Neil by, what she calls, “weapons of math destruction” (O'Neil, 2016). Her analysis of the health care insurance companies and their reliance on big data and AI algorithms demonstrated how those separate the sick from the healthy and create paths for debilitating inequalities in access to affordable health care.

The use of ADM systems in diagnosis is problematic for several reasons. Groups of patients who represent a minority in terms of some biological traits might find themselves systematically disadvantaged because the database used is insufficient for the respective group or leads to misjudgments (Algorithm Watch, 2019). Diagnostic tools, although very promising and receiving heightened attention in the last few years, are not yet deemed as safe and accurate enough to be used in everyday practise and their general uptake among clinicians is still low (Higgins and Madai, 2020). Secondly, the issue of trust in the use of ADM systems in health is critical. Consumers surveyed by MIT AGELAB indicated “little to some willingness to trust a diagnosis and follow a treatment plan developed by AI, allow a medical professional to use AI for recording data and as a decision support tool, use in-home monitoring on the health issues of their own, and trust an AI prediction on potential health issues and life expectancy” (MIT AGELAB, 2021). Also the medical practitioners are often sceptical or reluctant to rely on AI-delivered diagnosis (Allahabadi et al., 2022). Moreover, similar to the problems with non-representative datasets for training of machine learning models for face or emotion recognition discussed above, the datasets used for training diagnostic models also suffer from lack of proper representation in terms of age, as was shown in a study of a diagnostic model for detection of lung compromise in COVID-19 patients (Allahabadi et al., 2022).

With regard to negative consequences for ageing population and older adults, there is not yet enough systematically collected empirical evidence to illustrate how ADMs affect older adults on a group level. However, the evidence of discrimination in relation to many socio-demographic characteristics, many of which correlate highly with age, such as income, health status or employment status, might suggests that the disparate effect of ADMs used in health care sector on ageing populations might occur.

## 5. Final thoughts and discussion

Machines and technology have become integral parts of society and are shaping culture, civilisation and our general way of life today and in the future; it has virtually merged with our normative orientations and social models about ageing and older persons. AI technologies are certainly shifting how we will think about health, sickness, and ageing (Woods, 2020). And although technical and technological innovations trigger far-reaching consequences of how we perceive ageing (Wanka and Gallistl, 2018), the specific relationships between technology and power or the connection between technology and ageism has rarely been the focus.

An adequate and intersectional ethical approach is needed in design and development of AI, as well as in policy making, to safeguard that the algorithmic systems do not exclude and marginalised already vulnerable groups of older adults by neglecting social determinants of health. Currently, international efforts are being made in the realm of regulation of AI where guidelines and policy recommendations are drafted with regard to aspects of fairness, accountability, transparency in order to meet the criteria of trustworthiness of AI systems (e.g., currently debated European Artificial Intelligence Act). However, the outcomes and implementation of these regulations, particularly in the healthcare sector will pose additional challenges which are yet to be seen.

The large global campaign of World Health Organisation (WHO) to combat ageism (WHO, 2021) recognises the problem of IT sector as the one where ageism hits very hard. Moreover, the recent WHO Policy Brief titled “Ageism in artificial intelligence for health” investigates the use of artificial intelligence in medicine and public health for older people, including the conditions in which AI can exacerbate or introduce new forms of ageism (WHO, 2022). The policy brief stipulates that to “ensure that AI technologies play a beneficial role, ageism must be identified and eliminated from their design, development, use and evaluations” (WHO, 2022, p. 10). It proposes eight considerations for safeguarding that AI for health is developed in an equitable manner: participatory design, age diversity in data science teams, age inclusive data collection, investments in digital infrastructure and digital literacy of older people and their caregivers, rights of older people to contest and consent, governance frameworks to empower older persons, increased research and robust ethics processes. With certainty these guidelines are a good starting point in developing an ethical and equitable approach to building AI for health. However, their implementation into greater debates on bias in AI, as well as practical integration into the workflows of AI developers will require concerted efforts of the whole ageing research community, and far beyond.

Considering the recent revolutions in the development in AI and machine learning, it has become clear that technology is far more than a medium or a mere artefact that benefits all people to the same extent. The inequality-generating aspects of new technologies cannot be overlooked, nor can the fact that different technologies are always central resources for the exercise of power, that technical dominance has become logically inscribed in social structures and has become synonymous with influence and power. The report of MIT AGELAB (2021) concluded, that despite a relatively optimistic outlook on the capabilities and adoption of AI systems, the experts interviewed about the benefits of AI for ageing population were least confident in AI's ability to provide more equitable access to health care. They added “any system that replaces a human with an algorithm has the potential of making incorrect decisions that can threaten human health. Because health care literally involves life and death decisions, it is critical to build in enough redundancy and resilience in AI-based systems to ensure that these systems do no harm” (MIT AGELAB, 2021, p. 35).

Moreover, it is being increasingly debated that AI in medicine is viewed as overly positive and optimistic as to the capabilities of this technology in preventing or curing diseases. In fact, there are only few certified and even fewer clinically validated products available in the clinical setting. Most of the hype around medical uses of AI is related to cases of technology in the exploratory stages of development (proof of concept), which identify potentially valuable use cases, but which are yet to be validated in the clinical use trials (Madai and Higgins, 2021). Hence, many experts suggest caution in estimating the real effects of this technology on the future of healthcare for older adults (Berisha and Liss, 2022; WHO, 2022).

In addition, in view of the variety of AI techniques and their application for health, it is necessary to empirically examine them more closely and to ask what AI means in the respective fields of investigation and in which hidden patterns of age discrimination are integrated. Thus, it must be transparent where artificial intelligence is used and which influencing factors play a role. It must be possible to object to the use at any time.

This short contribution is by no means exhaustive of the topic and may just serve as a pointer in the direction of future research or critical thinking about the use of AI systems in heath and the way this might impact older adults.

## Data availability statement

The original contributions presented in the study are included in the article/supplementary material, further inquiries can be directed to the corresponding author.

## Author contributions

JS has developed the idea of the paper, the outline, and wrote parts 2, 3, and 4. AF participated in drafting the part 1 and 5 of the paper. Both authors contributed to the article and approved the submitted version.
